# Tumor-Homing Peptides as Crucial Component of Magnetic-Based Delivery Systems: Recent Developments and Pharmacoeconomical Perspective

**DOI:** 10.3390/ijms25116219

**Published:** 2024-06-05

**Authors:** Sylwia Milewska, Anna Sadowska, Natalia Stefaniuk, Iwona Misztalewska-Turkowicz, Agnieszka Z. Wilczewska, Halina Car, Katarzyna Niemirowicz-Laskowska

**Affiliations:** 1 Department of Experimental Pharmacology, Medical University of Bialystok, Szpitalna 37, 15-295 Bialystok, Poland; sylwiakowalewska1@gmail.com (S.M.); anna.sadowska@umb.edu.pl (A.S.); stefaniuk.natalia99@gmail.com (N.S.); halina.car@umb.edu.pl (H.C.); 2 Faculty of Chemistry, University of Bialystok, Ciolkowskiego 1K, 15-245 Bialystok, Poland; i.misztalewska@uwb.edu.pl (I.M.-T.); agawilczuwb@gmail.com (A.Z.W.)

**Keywords:** tumor-homing peptides, targeted delivery, targeting ligands, delivery systems, anticancer therapy, pharmacoeconomics, clinical analysis, cost-effectiveness analysis

## Abstract

According to data from the World Health Organization (WHO), cancer is considered to be one of the leading causes of death worldwide, and new therapeutic approaches, especially improved novel cancer treatment regimens, are in high demand. Considering that many chemotherapeutic drugs tend to have poor pharmacokinetic profiles, including rapid clearance and limited on-site accumulation, a combined approach with tumor-homing peptide (THP)-functionalized magnetic nanoparticles could lead to remarkable improvements. This is confirmed by an increasing number of papers in this field, showing that the on-target peptide functionalization of magnetic nanoparticles improves their penetration properties and ensures tumor-specific binding, which results in an increased clinical response. This review aims to highlight the potential applications of THPs in combination with magnetic carriers across various fields, including a pharmacoeconomic perspective.

## 1. Introduction

Nowadays, the use of peptides in cancer therapy has received increasing attention. On this basis, tumor-homing peptides (THPs) are now widely recognized as targeting molecules in drug delivery systems. With the advancements in phage display technology, many peptides that specifically adhere to cancer cells have been identified. These peptides are highly correlated with a specific type of receptor or marker that is frequently present in a number of tumors and tumor vasculature. In some cases, several of these receptors or markers were found to be overexpressed in tumors compared to their expression in normal tissues [1,2,3].

Furthermore, the ongoing development of drug delivery systems in nanomedicine, which could lead to effective cancer targeting connected with a high level of cellular internalization, is an essential goal for anticancer therapy. The combined use of therapeutic agents in nanoscale scenarios ensures an additive effect and enables the additional modification of the nanoparticle surface with targeting ligands for specific therapy of malignancies. To achieve this goal, there is a desire to develop a form of drug delivery to tumor cells with minimal side effects as compared to conventional chemotherapy [4,5,6,7,8,9]. Among many forms of carriers proposed in drug delivery systems (DDSs), iron oxide magnetic nanoparticles show great potential [10,11]. Magnetic core nanoparticles are highly attractive candidates in the biomedicine field, especially for targeting therapy, due to their high biocompatibility, ease of preparation, and ability to be surface-modified with various kinds of shells (metallic/polymeric/lipid) and functionalization (targeting molecules/fluorescent probes) as well as their optimal magnetic properties [12,13].

Worth noting is the fact that the economic burden of cancer treatment is a global problem. To minimize this effect, innovative approaches should be considered instead of standard chemotherapy, which has several side effects. To overcome these drawbacks, large-scale research has been conducted on the discovery, production, and optimization process of peptide drug conjugates [14].

From a pharmacoeconomic point of view, combining traditional peptide discovery techniques with cutting-edge technologies, such as rational design and phage display, ensures a fool-proof approach to designing effective and highly selective leading peptides in a short period of time. This modern therapy is undoubtedly important in the subsequent diagnosis and treatment of cancer by inhibiting tumor growth through drug accumulation in the affected area, resulting in lower direct and indirect costs of the process. Targeted and more efficient drug delivery is also essential for improving patient compliance and increasing the quality of life for patients with cancer [14,15,16].

This review summarizes the unique features and wide range of biomedical applications of nanoparticles combined with THPs, with special attention given to magnetic counterparts [16,17,18,19,20,21]. This review begins by discussing targeted receptors found on cancer membranes. The next section describes THPs in detail. Furthermore, theranostic aspects of tumor-targeting peptides will be discussed in this text, including drug delivery strategies and diagnostic applications, especially regarding magnetic nanoparticles. As numerous review articles have been published on the subject of targeted therapy using nanomaterials and homing peptides, this review will focus on the clinical and cost-effectiveness analysis of nanocarrier applications. At the end, future perspectives and conclusions will be presented.

## 2. Cancer Membrane—Targeting Receptors

The plasma membrane plays an essential role in many physiological processes, including drug transport. Its dynamics is crucial in the regulation of cell survival via all phases of the cell.

The basic structure of cell membranes, the lipid bilayer, is a substantial component of eukaryotic cell function, regulating loads of processes such as intracellular signaling, redox balance, and cell death [22,23]. These properties are due to the lipids’ aggregation capacity, forming highly dynamic and heterogeneous regions, known as lipid rafts. Lipid rafts are membrane microdomains (<200 nm) enriched in cholesterol and sphingolipids that selectively enroll specific protein receptors [24]. Alterations in the arrangement or composition of lipids result in various effects on cellular functions, affecting signal transduction, membrane movement, and its plasticity. Cytoplasmatic cholesterol is one of the most significant regulators of lipid organization, accounting for up to 90% of total cellular cholesterol [25].

**Figure 1:**
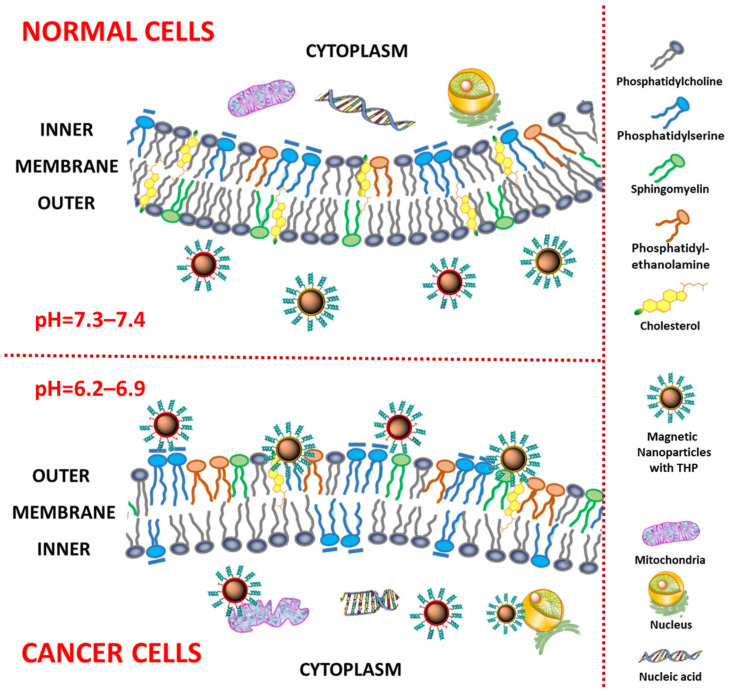
Differences between the membranes of healthy and neoplastic cells determine nanocarrier internalization.

Cell membranes in cancer and healthy cells exhibit distinct properties (Figure 1). Membrane attributes favor or inhibit drug penetration, conformation, and location, thus affecting therapeutic targets. Healthy cell membranes possess the asymmetric distribution of sphingomyelin and phosphatidylcholine in the outer leaflets and anionic phosphatidylserine and phosphatidylethanolamine in the inner leaflets [26], whereas in the cancer cell membrane, this asymmetric distribution and alterations in membrane fluidity are lost, which results in negative-charge phosphatidylserine exposure on the surface of the membrane, with the location of phosphatidylethanolamine on the outer leaflet [27,28]. Moreover, sphingomyelin is reduced in the cancer cell membrane, which is also associated with tumorigenesis [29]. Different lipid composition affects membrane fluidity, impacting drug penetration and biological action [30,31,32].

Extracellular acidity, with or without exosome release, can affect the pH level, which changes from 7.4 to 6.5, a characteristic pH level in cancer, and can contribute to the formation of the malignant tumor phenotype [33]. The acidic extracellular pH in the surrounding environment possibly promotes cancer invasiveness [34]. For rapid accommodation, cancer cells can reorganize their plasma membranes in order to retain proliferation, evade apoptosis, and maintain resistance against anticancer treatments [35].

It is expected that membrane–lipid therapy provides new treatments for various diseases, such as oncological and neurodegenerative diseases, stroke, and diabetes [25,36].

The majority of peptides with anticancer properties disturb cancer cells through apoptosis or necrosis [37,38,39]. Cholesterol, especially in high levels, is essential to prevent or inhibit lytic activity via the modification of membrane fluidity of eukaryotic cell membranes [40]. Membrane fluidity was noted to be higher in cancer cells in comparison to healthy cells. Moreover, cancer cells may have an increased surface area for absorption due to more plenteous microvilli.

In addition, healthy cells are electrically neutral, while cancer cells are negatively charged by their components on the surface, which promotes cytotoxicity, apoptosis, and the destabilization of the membrane after treatment by THP [41]. The interaction between peptides and the cell membrane is another difference. A healthy cell membrane and peptides make contact through hydrophobic interactions, while electrostatic interactions are involved in the interaction between peptides and the cancer cell membrane [32,42].

**Figure 2:**
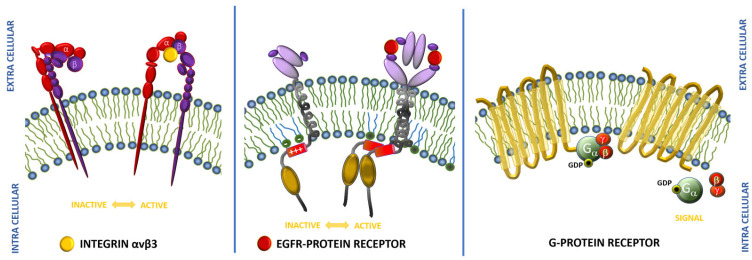
Schematic representation of main receptors interacting with tumor-homing peptides.

Targeted cancer therapy is a challenge in the development of novel anticancer drugs. Selective tumor targeting requires drug delivery strictly to the tumor site while sparing healthy tissue. Cell surface receptors provide the desired properties, such as ectopical overexpression in a high number on the malignant tissue in order to enable adequate selectivity. Therefore, the desired ratio of tumor to normal cell expression is 3:1 or higher. The number of overexpressed receptors should be sufficient to provide appropriate drug supply and achieve a suitable therapeutic effect [43]. Many proteins have been identified that are abundant on cancer cells compared to normal cells, including receptors such as integrins, epidermal growth factor receptor (EGFR), and G protein-coupled receptors (GPCRs). Schematic illustrations of the aforementioned receptors are presented in Figure 2.

Integrins are heterodimeric transmembrane receptors composed of two non-covalently connected transmembrane glycoprotein subunits, α and β. Integrins are significantly important as they are the main receptor proteins used by cells to bind to as well as to respond to the extracellular matrix (ECM). The linkage between the intracellular cytoskeleton and ECM allows for mediating the migration, proliferation, and adhesion of cells. Because all the aforementioned processes are crucial for carcinogenesis, from many different integrins, αvβ3 appears to be the most important target for anti-angiogenic cancer therapy, due to its association with angiogenesis and tumor metastasis. Integrin αvβ3 is overexpressed on activated endothelial cells, new-born vessels, and tumor cells, but is not found in resting endothelial cells and most adult epithelial cells [43,44,45].

Another transmembrane protein worth mentioning is the epidermal growth factor (EGFR), which belongs to the ErbB tyrosine kinase family. It is overexpressed especially in various cancer types, such as lung, ovarian, or breast cancer, and it is also linked to increased cancer cell proliferation. Therefore, the inhibition of EGFR and suppression of tumor growth is the target in clinical use of monoclonal antibodies and small-molecule inhibitors [46].

The largest class of transmembrane proteins comprising about 800 receptors is the G protein-coupled receptors (GPCRs). They possess an extreme pharmacological potency with about 15% of receptors targeted by FDA-approved drugs [47]. Proteins from this family mediate most cellular responses to hormones and neurotransmitters, and are responsible for vision, olfaction, and taste. GPCRs are often overexpressed in carcinogenesis, which allows the precise targeting of tumor cells with peptide conjugates [43]. These receptors also serve as attractive drug targets due to their significance in the treatment of other various diseases, like inflammatory and cardiac disorders, and metabolic imbalances [48].

Additionally, several other peptide receptors have been investigated as targets for antitumor drug delivery, and these include gonadotropin-releasing hormone receptor (GnRH-R), vasoactive intestinal peptide (VIP) receptors 1 and 2, melanocortin receptor 1 (MC1R), and neurotensin receptor 1 (NTSR1) [43].

## 3. Tumor-Homing Peptides (THPs)—Characteristics

An increasing number of researchers have focused on EGFR mutations, which are among the most commonly identified. Therefore, the safe and effective management of cancer treatment requires the implementation of some standard procedures, such as screening for predictive and prognostic biomarkers with targeted agents. Aforementioned tools could help predict sensitivity to targeted therapy and estimate survival rates for patients with carcinoma. Rationally designed tumor-homing peptides and/or cell-penetrating peptides with targeting ligands could be helpful in many areas, starting with the inhibition of particular mutations, leading to a more efficient and cost-effective clinical trial process [49,50].

THPs are classified as short peptides, consisting of 3–15 amino acids, that specifically identify and attach to cancer cells or tumor blood vessels. Since the introduction of the tumor-homing approach in 1998, various THPs have been identified using phage display techniques, both in in vitro and in vivo assessment. They can attract specific phenotypes of cancer cells and their environment due to their specificity. THPs contain several typical motifs like arginine–glycine–aspartic acid (RGD) and asparagine–glycine–arginine (NGR) peptides, which specifically bind to the molecules on the surface of cancer cells or tumor blood vessels [51].

The first peptide (RGD) is one of the most studied tumor vasculatures targeting ligands, while NGR is the second one. Both of them have potential applications in antitumor therapy and drug delivery [52].

### 3.1. RGD Peptides—Characteristics

The RGD peptide targets tumor vessels that exhibit a selective expression of αvβ3 and αvβ5 integrins [51,53]. These integrins are known to be overexpressed in tumor-associated blood vessels and glioma cells, and their activation initiates pathways that are associated with cell proliferation and tumor-induced angiogenesis [53]. Integrins play a crucial role in regulating intracellular signaling pathways that protect cancer cells from the antiproliferative effects of anticancer drugs. The distortion of this protein could be useful in sensitizing tumor cells to antitumor agents [53].

### 3.2. Cyclic RGD—Characteristics

These integrins are known for their specific properties, and they are expressed on tumor cells and are responsible for activating endothelial cells in tumor neovascularization. The correlation levels of the αvβ3 integrin with tumor growth progression and aggressiveness make an attractive biological candidate for the development of antiangiogenic drugs and molecular imaging for early cancer diagnoses. The cyclic RGD peptide can be mono-, di-, or multimeric and is commonly used as a radiotracer or targeted biomolecule to transport the αvβ3 and αvβ5 isotope integrins. The radiolabeled properties of cyclic RGD are especially useful for radiotracking while imaging with PET/SPECT methods. The radiolabeled cyclic RGD peptides are also able to bind to other integrins, for instance, αvβ5, α5β1, α6β4, α4β1, and αvβ6, which lead to an increase in the tumors’ uptake by enhancing the receptor population [54,55,56].

### 3.3. iRGD—Characteristics

The iRGD peptide, recently discovered through phage display, is a new peptide that can be used for tumor targeting. It has a short amino acid sequence (Arg-Gly-Asp) and it is used to monitor tumor cell permeability, regulate cell internalization and extravasation, and promote further tissue penetration to enhance imaging sensitivity and therapeutic effectiveness [57,58,59].

The iRGD peptide possesses many advantages. Similar to the conventional RGD peptide, iRGD (CRGDK/RGPD/EC) can bind to αv integrins after i.v. administration, which are specifically expressed on the surface of tumor vessels. The iRGD is then cleaved to CRGDK/R through a protease. Since iRGD has a functional sequence, it shows affinity for NRP-1, particularly the active C-end Rule (CendR) motif (R/KXXR/K) exhibited at the C-terminus [57,60,61,62]. The penetration of tumor tissue is induced by binding of the peptide to integrins via CendR motif interaction with NRP-1. This binding process enables the extravasation and permeation of imaging agents or drugs that are conjugated to the iRGD peptide or administered together with iRGD into target tissues and tumor cells [57,59,63,64]. Moreover, iRGD exhibits better biological homing properties, probably due to the specific homing of the intact peptide by RGD. Furthermore, the iRGD recruitment to the cell surface via iRGD–integrin interaction is presumably required for proteolytic cleavage, which triggers the subsequent tumor penetration, as protease inhibitors are typically nonviable at cell surfaces but can block proteolysis elsewhere [57,60]. It should also be emphasized that the ability of iRGD to penetrate cells is much better compared to conventional RGD peptides, probably due to the fact that integrins move between the cell surface and intracellular compartments, and several viral pathogens can penetrate cells using this mechanism [65]. Higher tumor apoptosis and anticancer efficiency were observed due to the concomitant effect of EPR (enhanced permeability and retention) and the mediating effect of active targeting of iRGD [66]. Conclusively, published results indicate that iRGD does not show negative effects or cytotoxicity against healthy cells, which increases its potential as a homing molecule [67].

### 3.4. NGR Peptides—Characteristics

The NGR motif is a homing tripeptide that binds to Aminopeptidase N (APN or CD13), which is overexpressed on the surface of many cancer cells [51,53]. In effect, these sequences recognize and bind CD13 on tumor endothelial cells. Moreover, the deamidation of the NGR motif provides an additional recognition moiety called Iso-DGR, which specifically targets αvβ3 integrins expressed on tumor neovasculature [68]. The receptors mentioned above are known as integral parts of angiogenesis regulators and are overexpressed on the endothelium of solid tumors where they are responsible for the promotion of tumor vascularization [69]. Other reports indicated that αvβ3 integrins play a key role in metastasis. Interesting is the fact that these receptors are only expressed in newly formed vessels [70]. Recent studies have shown excellent properties for inhibiting cancer growth. The NGR peptide could also be used as a vehicle for delivering nanoparticles, chemotherapeutics, and radioisotopes to tumors [70]. An in vivo and in vitro assessment showed that an NGR peptide radiolabeled with 99mTc demonstrated great potential as a diagnostic agent, especially in the visualization of lung tumors [53,70]. In effect, NGR targeting properties are crucial for developing a method for non-invasive imaging of CD13 receptor levels in living individuals, which may help to identify treatment-responsive patients, monitor therapy, and assess prognoses. Molecular radiological imaging based on NGR is the most effective method to assess CD13 receptor expression in vivo in a non-invasive and accurate manner [70].

### 3.5. Cell-Penetrating Peptides (CPPs)—Characteristics

Cancer cells can be differentiated from healthy cells by their higher concentration of negatively charged cell surface compounds, including negatively charged head groups of the lipid bilayer that forms phospholipids. This favors them to interact with cationically charged peptides and is partially responsible for the relative selectivity of cancer cells [32,71,72].

CPPs are classified as short peptides that have the ability to penetrate tissues and cell membranes in an energy-dependent or energy-independent manner. They are used to transport a diverse range of bioactive conjugates (cargoes) inside cells, including peptides, proteins, DNA, siRNAs, small drugs, NPs, fluorescent agents, and more. CPPs are highly advantageous due to their biocompatibility and the ability to modify peptide sequences to fine-tune hydrophobicity, charge, affinity, stability, and also solubility. Additionally, they can be easily synthesized in adequate quantities [18,73,74].

CPPs can be categorized based on their origin, role, or sequence, or due to their mechanism of uptake. According to their physicochemical properties, they can be classified as amphiphilic, cationic, or hydrophobic [73].

At physiological pH, cationic CPPs have a high positive net charge. They are derived from basic short-chain arginines and lysines, such as TAT48-60 (GRKKRRQRRPPQ) or DPV1047 (VKRGLKLRHVRPRVTRMDV). Hydrophobic CPPs are primarily composed of apolar moieties that contain amino acid groups important for cellular uptake, combined with a low net charge, for instance, C105Y (CSIPPEVKFNKPFVYLI). The amphiphilic CPPs are the third class of CPPs. They contain both hydrophilic and hydrophobic regions of amino acid sequences. The typical examples are pVEC (LLIILRRRIRKQAHAHSK) and Pep1 (KETWWETWWTEWSQPKKRKV). According to their origin, peptides can be categorized as chimeric CPPs, CPP derivatives, and synthetic CPPs [73,75].

Cell-penetrating peptides (CPPs) are highly promising ligands, drawing the attention of researchers based on their efficiency in transporting bioactive molecules intracellularly. However, the lack of specificity and their in vivo degradation have led to the development of more recent types of CPPs. Currently, activated CPPs and modified tumor-targeting peptides (TTPs) show much better specific cellular uptake, cytotoxicity, and inhibition of tumor growth [43,73].

Although CPPs have huge potential as carriers for specific drug delivery into tumors, the majority of conventional CPPs have several limitations, including a deficiency of cell specificity and in vivo instability, which are leading barriers to their future therapeutic development. There are limited in vivo applications of CPPs due to their non-specific tissue/cell penetration. Several approaches have been proposed so far to selectively target tumor cells with CPPs. In order for CPPs to be useful as vectors for delivering therapeutic agents, their in vivo stability must be considered. Due to their peptide nature, CPPs are susceptible to degradation by proteases both extracellularly and intracellularly. The stability of CPPs in vivo is determined by various factors that affect proteolytic cleavage, such as the amino acid sequence, conformation, chemistry, administration routes, conjugate cargo type, and method of CPP—cargo conjugation [73].

### 3.6. Machine Learning Approaches for Designing THP

In general, for designing new drug targeting devices, three main routes are implemented: 1. the structure-based drug design (SBDD), 2. the ligand-based drug design (LBDD), and, if the protein structure is not known, 3. computational methods are used (amongst them are those based on machine learning) [76]. Although experimental approaches can facilitate the precise identification of THPs, they are usually labor-intensive, time-consuming, and expensive. For those reasons, nowadays, more and more computational methods (including machine learning ones) are used to predict the structure of THP. To date, three webservers based on machine learning models are currently active, the TumorHPD and the THPep, and more recently the SCMTHP. The TumorHPD not only facilitates the THP prediction but is also able to design THPs with better tumor-homing properties. This system firstly generates all possible single substitution derivatives of an original peptide; then, it predicts if the original peptide and the derivative is THP or non-THP [77]. The THPep is a sequence-based approach for the prediction and analyzing of THPs by using an interpretable random forest classifier in accordance with amino acid composition, dipeptide composition, and pseudo-amino acid composition. The authors pointed out that the precise identification and efficient characterization of tumor-homing peptides based on a computational model is crucial to understand their role in the drug development process and also to reduce the time and cost of tumor-homing peptide-based therapy [78]. After conducting a range of tests, including rigorous cross-validation using 5-fold cross-validation, it was found that the proposed model of THPep is incredibly powerful and holds great promise for use in basic research and drug development. Based on aforementioned research, a free webserver was built to provide an efficient and useful tool for THP prediction: http://codes.bio/thpep/ (accessed on 23 May 2024) [78]. The SCMTHP approach is a simple and easily interpretable computational system, which is using the scoring card method (SCM) for identifying and analyzing tumor-homing activities of peptides [79]. Another novel example of a machine learning-based framework is StackTHPred that predicts THPs using optimal features and a stacking architecture, and facilitates the identification of THPs from complex samples [80]. Next, machine learning-based methods distinguish THPs from non-THPs by encoding peptides, selecting optimal features, and finally identifying THPs by a support vector machine (SVM) [81]. A novel computational approach for THP named NEPTUNE was presented by Charoenkwan et al. It facilitates the accurate and large-scale identification of THPs from sequence information. This approach includes six popular machine learning algorithms from which information is fed into an SVM-based classifier (meta-predictor NEPTUNE) [82]. Another method based on network science and similarity searching implemented in the starPep toolbox was described by M. Romero et al. This method employs Chemical Space Networks for extracting the most relevant and non-redundant THP sequences, which are used in multi-query similarity searching models (SSMs) [83].

## 4. Homing Peptides for Drug Delivery

### 4.1. THPs for Drug Delivery

Drug-targeting peptides have been widely examined as homing molecules in drug delivery systems due to their specific binding properties and ability to efficiently accumulate drugs at the site of action. However, they still pose a challenge for large-scale chemotherapy-related research in cancer treatment. Indeed, through the chemical linking of the ligand of interest to drug-loaded nanocarriers, active targeting systems could be developed. Peptide-functionalized nanostructures have found broad application in various biomedical fields, encompassing drug delivery, bioimaging, fluid biopsy, and target-based anticancer therapies. They are well recognized as potential new therapeutic candidates, owing to their high specificity, excellent biocompatibility, and ease of delivery [84,85].

Biomolecules including tumor-homing peptides can be conjugated with the drug delivery system’s core in two general ways: by non-covalent and covalent bonding. Non-covalent bindings are represented mainly by π–π stacking forces, hydrophobic interactions, electrostatic interactions, and hydrogen bonds. Preparing the delivery system based on non-covalent interactions does not demand the application of complicated and tough synthetic methods. However, they are weaker and less stable in their nature than covalent bonds, which can limit their application. The most popular covalent bonds applied for THP’s conjugation are amide and ester bond preparation (using variety of bond activation strategies—e.g., NHS ester formation) and the Michael reaction of an amine group (mainly by maleimide derivative preparation), though such binding may affect the bioactivity of peptides. The examples of the bonding strategies are presented in Table 1 below:

It has been a goal of many researchers to target specific cell types to achieve an optimal biodistribution of therapeutic agents in affected tissues, while reducing potential side effects.

Many papers have discussed the use of peptide-specific tissue biomarkers that enable peptides to act as homing devices for targeting specific tissues or organs. Such properties are especially characterized by homing peptides and cell-penetrating homing peptides. In addition to enhancing cellular uptake, these peptides can also act as homing ligands for various carriers and compounds, such as nanoparticles or drugs, to enter cells [68,69].

With reference to the above, we have focused on the advantages of using various types of nanoparticles functionalized with targeting ligands (Table 2).

Summarizing this part, it should be emphasized that the targeting of tumor cells or tumor blood vasculature using THPs has received considerable attention in the past few years. The most commonly used homing peptides for drug delivery to cancer tumors include RGD and NGR. There are THPs that precisely adhere to receptors overexpressed on cancer cells, both through phage display methods and an appropriate design and modification of natural peptide ligands [100,101,102,103]. Nanomedicines can be targeted to particular tissues using homing peptides in order to improve exposure to therapeutic agents. Indeed, these large targeting nanopeptides can accumulate in the targeted tissues to a greater extent than small conjugates, hence improving therapeutic efficacy and minimizing side effects. Furthermore, some groups have coupled tumor-homing peptides with imaging particles, including fluorescent probes and positron emission tomography or magnetic resonance imaging (PET or MRI) molecules, to enable migration to disease sites, as presented in Table 1 of this paper [18,52,104].

Nanoforms, such as polymers, micelles, and liposomes, may serve as drug carriers to improve targeting efficacy by increasing circulation time. Clinically, the application of nanoparticles based on albumin, liposomes, or polymers encapsulated with anticancer drugs has been demonstrated to have superior therapeutic efficacy, improved stability, enhanced solubility, and reduced toxicity compared to each of the individual free drugs. The benefits of using nanoparticles are also reported in numerous scientific papers [105,106,107,108,109,110].

Nanoparticle conjugation with various ligands has exhibited promising possible therapeutic effectiveness in targeted drug delivery. Achieving the additional vacation and deep penetration of antitumor nanoparticles into the tumor mass seems to remain a challenge that could possibly be avoided through the use of tumor-penetrating peptides [111]. It should also be noted that in all approaches, the decoration of NPs with CPPs and/or targeting peptides led to an improved cellular uptake, indicating an effective combination of these units [101,112].

Thus, it was proved that the addition of a tumor-penetrating peptide to the surface of nanoparticles can enhance their specific properties such as cancer selectivity, tissue penetration, and potency [113,114].

### 4.2. THPs for Blood–Brain Barrier (BBB) Delivery

Theranostics, defined as an integrated system for a diagnosis, targeted therapy, and response monitoring, may be an attractive strategy for brain cancers. Despite the blood–brain barrier (BBB) representing a significant challenge to drug delivery, the field of nanomedicine has identified promising avenues for the efficient delivery of chemotherapeutics across the BBB and into brain cancer sites, while simultaneously reducing adverse effects on healthy tissue. In recent years, peptides have emerged as excellent candidates both for crossing the BBB and for homing in on specific targets in brain tumors. They are small and very specific, exhibiting remarkable sequence flexibility and genetic/chemical conjugation to other molecules, such as nanoparticles of diverse origins. These conjugates can then be employed as drug delivery vehicles for specific chemotherapeutic applications [115,116].

Three stages of brain tumors have been identified as relevant when designing drug delivery systems. In the first stadium, when tumor formation has occurred but the BBB is intact, it is important to target nanoparticles that can cross the integrity of the BBB and transport the cargo to the tumor site. In the second stage, tumor cells are dividing rapidly and have already induced the secretion of EGF and the expression of VEGFR and EGFR receptors, which promote angiogenesis. This results in a permeable or defective BBB, allowing the passage of higher-molecular-weight molecules. The third stage is when a blood–brain tumor barrier (BBTB) is created. In such cases, strategies to target angiogenesis via peptides such as RGD or nanocarriers with a size of less than 12 nm that can pass through negatively charged pores on the BBTB should be applied [117,118]. Consequently, the treatment of brain cancer (such as glioblastoma) presents a challenge to the use of peptides that address issues such as BBB crossing, cell penetration, and specific glioma homing. Guiding peptides can be conjugated directly to a drug or serve as the directing component of a complex therapeutic system that contains a drug encapsulated in a carrier. Carriers that have been applied in the treatment of tumors located in the brain include liposome or polymeric-based systems such as methoxy-PEG and PEG-PLA. Examples of peptides along with carriers and ligands in the treatment of glioblastoma are summarized in Table 3.

## 5. Homing Peptides for Imaging

THPs could be adapted as diagnostic tools with greater efficiency, providing entirely new opportunities in tumor detection. Moreover, due to their properties and simplicity of modification with functional groups, THPs could be applied as multifunctional imaging platforms, especially including radionuclide, optical, ultrasound, and magnetic resonance imaging techniques [134].

Appropriate and early cancer detection, as well as effective prevention, is one of the main goals of effective anticancer therapy. Nowadays, computed tomography (CT) and magnetic resonance imaging (MRI), which are classified as conventional imaging techniques, can detect cancer when the tumor is larger than one centimeter in diameter. Therefore, it is clear that more responsive imaging modalities are required for the early detection and improved efficiency of tumor identification. Conventional imaging techniques are based on the structure of anatomical organs, while molecular-based imaging can recognize site-specific molecular probes and use certain receptors uniquely in cancer management. Molecular imaging is claimed to be a potentially promising approach for the early screening and identification of changes in crucial morphological behaviors and local host responses associated with the early stages of specific incidents in the disease spectrum, including progression at the cellular level [135,136].

Therefore, tumor-specific homing peptides with high binding affinity are a promising diagnostic technique for targeted cancer imaging in a specific way. THPs have been examined in several imaging modalities such as molecular imaging inquires. Both RGD and NGR peptides have been commonly applied to transport various imaging agents [137,138].

A recently published paper [52] demonstrated several examples of peptide-conjugated nanocarriers used for in vivo cancer diagnostics. The author assessed 13 targeting peptides and their conjugated nanocarriers and showed their imaging properties. In most cases, peptide-conjugated nanocarriers presented MRI and optical features. The author also pointed out the advantages of using peptide-directed nanomedicine. First of all, an in vivo assessment revealed that an activated tumor endothelium could be imaged using both methods; however, only the optical technique can visualize angiogenic blood vessels and lymphatic vessels in tumors. Secondly, MRI imaging showed that nanoparticles can penetrate blood vessels and accumulate in tumor vessels in cases of breast cancer. What is more, this technique is also helpful in the in vivo assessment of HER2-overexpressing ovarian cancer cells.

The circulation time or half-life (T1/2) of nanostructured agents is a parameter of great importance for in vivo experiments and other clinical procedures [139,140,141]. Monitoring of biodistribution and clearance in vivo is also essential in this field [142]. Discussed properties are mostly controlled by the intrinsic properties of NPs, including the surface charge, shell material, size, and core, which directly affect the circulation time and the target area [142,143].

## 6. Magnetic Nanoparticles as a Platform for Tumor-Homing Peptides

### 6.1. Methods of Preparation and Functionalization of Magnetic Nanoparticles

The most commonly synthesized magnetic nanoparticles for biomedical applications are iron oxide nanoparticles (IONPs) consisting of magnetite (Fe_3_O_4_), maghemite (γ-Fe_2_O_3_), hematite (α-Fe_2_O_3_), or mixed ferrites [144]. The popularity of IONPs as drug carriers comes from their biocompatibility, non-toxicity, ease of preparation, and stability [145,146,147].

The synthetic routes for IONPs can be divided into three categories: chemical synthesis, biosynthesis, and physical methods. The most commonly used methods are the chemical ones, which represent around 90% of methods described in the literature [148]. The most efficient chemical methods are co-precipitation, sol–gel, thermal decomposition, and micro-emulsion [149,150].

**Figure 3:**
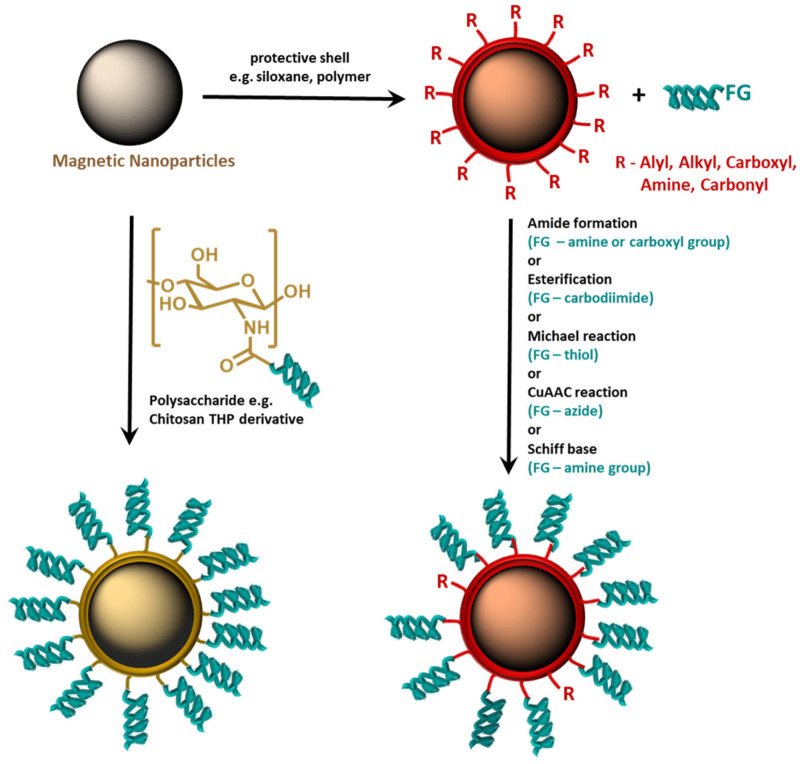
Anchoring of tumor-homing peptides on the surface of magnetic nanoparticles.

In the literature, several THP conjugation approaches to magnetic nanoparticles are implemented (Figure 3). Most of them are based on MNP coating with a biocompatible shell (PEGs, polysaccharides), followed by a reaction with the peptide or peptide derivative. The reactions most commonly used to anchor THPs onto nanoparticle surfaces are

- The esterification reaction of a carbodiimide THP derivative [151,152];
- The formation of an amide (on free NH_2_ [153] groups or –COOH [154] groups presented on the surface of MNP);
- The Michael reaction of a peptide’s SH group with meleimido-modified MNP [155,156,157,158];
- Copper Catalyzed Azide Alkyne Cycloaddition (reaction of alkyne-modified MNP with azido derivative of polypeptide) [159];
- Schiff base formation between a carbonyl group presented on the surface of MNP and an amino group of the THP [160].

Also, a different approach is implemented when using a THP-modified coating agent to build the active shell around MNP. Usually, polysaccharides are used as effective coating agents and good materials for THP derivative synthesis [161,162].

Gaining insight through the complex interactions between homing ligands and NPs and identifying appropriate coupling mechanisms to ensure adequate stability and biocompatibility with no degradation of peptide functionality are major challenges for successful clinical application. However, while many efforts have been undertaken to design smart multi-modal signal generating vectors, the growing complexity of the prepared nanoforms presents a challenging and in many cases labor-intensive assignment. Therefore, a key challenge is to establish reproducible synthetic protocols. Accordingly, there is a great need for straight single-pot synthetic pathways providing functional delivery of nanoplatforms that could be associated with new and smart linking methods to ensure colloidal stability under physiological conditions [163,164,165,166].

### 6.2. Biomedical Application of Magnetic Nanoparticles

Magnetic nanoparticles (MNPs) are widely known for their multifunctional properties in many areas of application. In particular, in terms of biomedical applications, they have been used for the detection and separation of cells, especially in stem cell tracking and signaling. In addition, they are used as therapeutic agents for imaging and hyperthermia and also as drug delivery systems. This wide range of capabilities is a very unique factor that enables their application in terms of diagnoses and therapy as well, resulting in a variety of application possibilities [150] (Table 4, Table 5, Table 6 and Table 7).

#### 6.2.1. MNPs in Cancer Imaging

Table 4 presents a few examples of peptide-conjugated magnetic-based nanocarriers used for cancer imaging.

**Table 4:** Peptide-conjugated magnetic-based nanocarriers for cancer imaging.

Imaging Method	Targeting Peptide	Nanocarrier	Advantages	Ref.
MRI	LTVSPWY	LTVSPWY-PEG-CS MNPs	In vivo assessment of HER2-overexpressing SKOV-3 carcinoma cells Effective ability to target and internalize cells, resulting in enhanced contrast images that are highly informative	[161]
	A54	A54-GFP-coated MNPs	Superparamagnetic properties at room temperature In vitro bioassay showed that coating with a homing peptide gives MNPs a specific affinity for cancer cells Possesses magnetic, cancer cell-specific, and fluorescent properties Promising tool in magnetic cell separation and purification	[151]
		A54-Dex-PLGA/DOX/SPIO	Both in vitro and in vivo assays showed the antitumor efficacy to BEL-7402 hepatoma cell line Less toxic in comparison to thypical adriamycin injections Promising tool in cancer binding and targeting	[162]
	iRGD	iRGD-SPIO	Improvement in the sensitivity of pancreatic cancer imaging Enhancement in the positive labeling rate of cells and the SPIO absorption Promising tool in cancer targeting and tissue penetration	[67]
	CKAAKN	CKAAKN–HA–VES@USPIO NPs	Improvement in tumor-targeting delivery of MRI contrast agents Superior specificity in the detection of pancreatic cancer Exquisite biosafety Over 80% of cell viability in BxPC-3 and HPDE6-C7 cell lines	[167]

Abbreviations: A54—AGKGTPSLETTP peptide, A54-Dex-PLGA—A54 peptide-functionalized poly(lactic-co-glycolic acid)-grafted dextran with encapsulated doxorubicin, CKAAKN–HA–VES@USPIO—peptide-functionalized amphiphilic hyaluronic acid–vitamin E succinate polymer (CKAAKN–HA–VES) for delivering ultra-small superparamagnetic iron oxides, iRGD—9-amino acid cyclic peptide (sequence: CRGDKGPDC), MNPs—magnetic nanoparticles, MRI—magnetic resonance imaging, NPs—nanoparticles, PEG-CS—PEGylated chitosan, SPIO—superparamagnetic iron oxide. Furthermore, peptide-conjugated magnetic-based nanocarriers have been successfully applied as other imaging methods.

Luo and coauthors [168] described various types of magnetic nanoparticles used in stem cell therapy. For instance, optical methods using luciferase substrates, fluorescent protein tags, fluorescent dyes, and near-infrared fluorophores enable the simultaneous assay of multiple cell lines and can be used in combination with other imaging modalities. Whole-body 3D scanning is a promising method that enables single-cell detection and is known for no ionizing radiation effects. PET and SPECT are two other diagnostic methods, and PET is known for its high-energy positron emitters, while SPECT enables high-energy gamma emitters with high detail. Ultrasound provides single-cell detection without ionizing radiation and soft tissue imaging. This method is also relatively inexpensive and fast. Table 5 presents a few examples of peptide-conjugated magnetic-based nanocarriers used for cancer diagnoses.

**Table 5:** Peptide-conjugated magnetic-based nanocarriers for cancer diagnostics.

Diagnostic Method	Targeting Peptide	Nanocarrier	Advantages	Ref.
Magnetic field	YSA	MNPs-YSA peptide conjugates	Extensive removal of spreading ovarian cancer cells from the abdomen can lead to a reduction in the number of malignant cells and decreased chances of metastatic spread Promising tool in cancer targeting and removal	[169]
Whole-body imaging	CREKA	CREKA-SPIO	Depletion of RES macrophages in the liver with liposomal clodronate The i.v. injection of Ni-liposomes prolonged the half-life of the nanocarrier Induction of blood clotting in tumor vessels Clotting amplification to tumor targeting Promising tool in improving the tumor detection by microscopic and whole-body imaging methods	[170]
Sensitive monitoring of the magnetic relaxation of IONPs with the use of MPS		IONPs-N/IONPs-N-P/IONPs-N-P with protease	Helpful method in detecting specific proteases Promising tool in diagnosis and treatment of cancers, Alzheimer’s disease, and vascular diseases	[171]
MRI	A54	Dex-PLGA/DOX/SPIO	Specific binding ability of A54-Dex-PLGA/DOX/SPIO micelles to hepatoma cell BEL-7402 Better therapeutic effects and reduced toxicity compared with commercial adriamycin injection	[162]
MRI	-	MNPs	The in vivo imaging of cells is an essential point in tracking and monitoring the treatment process, distribution of immune cells in the body, time of diffusion, proliferation, and migration rates MRI for in vivo imaging studies is an excellent method used for high-resolution soft tissue penetration MRI is used to show the exact internal structure of the body due to the magnetic field, radio waves, and electric fields MNPs lower the relaxation period of surrounding protons, making them good candidates for MRI contrast agents	[172]

Abbreviations: IONPs—iron oxide nanoparticles; IONPs-N—iron oxide nanoparticles–neutravidin; IONPs-N-P—iron oxide nanoparticles–neutravidin-peptide; MPS—magnetic particle spectrometer; MNPs—magnetic nanoparticles; MRI—magnetic resonance imaging; NPs—nanoparticles; i.v.—intravenously; PLGA—poly(lactic-co-glycolic acid); Dex—dextran; DOX—doxorubicin.

#### 6.2.2. MNPs in Hyperthermia Treatment

Hyperthermia is classified as an adjuvant therapy for cancer that uses temperatures higher than the physiologically optimal range, typically 40–43 °C. The duration of the treatment is approximately one hour [173]. Hyperthermia therapy can be successfully applied as a sensitizer before radio- and chemotherapy or as a component of a synergistic treatment [174]. To date, hyperthermia methods have demonstrated efficacy in the treatment of various types of cancer, including soft tissue sarcoma, melanoma, head and neck cancer, bladder cancer, and breast and cervical carcinoma [175]. During hyperthermia therapy, stability, function, and properties of cellular components, as well as cellular responses, including DNA repair pathways and systemic immune responses, have been altered. Moreover, hyperthermia may further help in the mitigation of chemo-resistance to doxorubicin in certain cancer cells [176].

From a historical perspective, the experiment conducted by Roizin-Towle et al. demonstrated that the survival of normal and neoplastic human cells exposed to a specific thermal dose could be decreased [177]. Nevertheless, tumor cells did not exhibit significant sensitivity to the treatment compared to healthy cells. This study suggests that the therapeutic success of hyperthermia will be contingent upon the ability to localize heat specifically in tumor cells. To achieve the aforementioned approach, the use of magnetic nanoparticles (MNPs), which are characterized by unique physicochemical properties and morphology, will be justified as hyperthermia agents [178]. The specific size range (10–150 nm) and the possibility of the surface functionalization of MNPs via homing molecules, in addition to the presence of leaky vasculatures around the cancer environment and the molecular profile of cancer cells, facilitate the delivery of nanomaterials to the tumor site. Magnetic nanoparticles may be employed to induce hyperthermia, specifically in cancer cells, through the application of alternating magnetic fields. Moreover, the incorporation of temperature-sensitive structures with a magnetic core can enhance the distribution and concentration of drugs in tumors, thereby facilitating the development of combined therapies. The examples of magnetically induced hyperthermia in cancer therapy are presented in Table 6.

**Table 6:** Peptide-conjugated magnetic-based nanocarriers for hyperthermia treatment.

Carrier	Ligand	Agent/ Tag	Tumor	Result	Ref.
Magneto-liposome	cRGD	DOX/ICG	In vitro: lung, breast, skin, brain, and liver cancer In vivo: BALB/c mice murine immuno-competent fibrosarcoma tumor model	Combinatorial tumor therapy (chemo-radio-hyperthermia) Insignificant cardiac toxicity	[179]
Fe_3_O_4_@PMAO-PEG	RGD	ND	In vitro compatibility assay: Vero cells	Prototype system for further in vivo evaluation	[180]
Fe_3_O_4_@PMAO	RGD	ND	In vivo: rats bearing hepatic implants of colon adenocarcinoma	Therapeutic approach for poorly vascularized liver tumors	[181]
Fe_3_O_4_	EGFR— targeted peptide (YHWYGYTPQNVI)	ND	In vitro: lung cancer (NSCLC) In vivo: mouse orthotopic lung tumor model	Effective anticancer treatment modality for the treatment of NSCLC based on targeted magnetic hyperthermia	[182]
TMNPs, i.e., Fe_3_O_4_@Mn_0.5_Zn_0.5_Fe_2_O_4_@CoFe_2_O_4_	LN1 CPP	ND	In vitro: prostate cancer	Reduction in cancer cell aggressiveness	[183]
SPIONs-PEG	membranotropic peptide gH625	Cyanine 5.5	In vitro: breast cancer cells	Prototype of nanoplatform for cancer theranostics involving magnetic resonance imaging, optical imaging (infrared), drug delivery, and hyperthermia	[184]

Abbreviations: PMAO—poly(maleic anhydride-alt-1-octadecene); NSCLC—non-small cell lung cancer; TMNPs—trimagnetic nanoparticles; ICG—indocyanine green; ND—not determined.

#### 6.2.3. Different Biomedical Applications of MNPs

Table 7 presents a few examples of biomedical applications of peptide-conjugated magnetic-based nanocarriers, which are used for treatment of life-threatening diseases such as cancer or bacterial infection.

**Table 7:** Different biomedical applications of peptide-conjugated magnetic-based nanocarriers.

Condition	Targeting Peptide	Nanocarrier	Advantages	Ref.
Conditions associated with Gram(+)/Gram(−) bacteria	Gly-Ala-Phe-Pro-His-Arg	Silica-coated iron oxide NPs	Internalization into *E. coli* and *S. aureus* bacterial cells Great improvement in the antibacterial effect with low doses of VAN Rapid process of targeted drug delivery Reduction in drug dose Equal effectiveness against both Gram(+) and Gram(−) bacteria	[185]
GBM	NFL peptide	pSiNRs	Effective administration Improvement in treatment Facilitated targeting Enhanced internalization Preferential uptake Promising tool in the treatment of brain tumors	[186]
ALI	iNOS PNAs and CPPs	SCKs	Promising tool in the design of hierarchical nanostructures with great potential to become antisense imaging agents and therapeutics	[187]
LC	TAT-functionalized IONPs	Fe_3_O_4_ + TAT	Increased cellular uptake Lysosomal destabilization following internalization Increased cellular ROS generation upon exposure to an alternating magnetic field Mitochondrial integrity in cell lines Increased cell apoptosis	[188]
UTIs	rGO/MPND with pyrene–PEG	rGO	Rapid capture and efficient elimination of *E. coli* Total ablation of *E. coli* after irradiation of the loaded nanocomposite with a near-infrared laser Promising method for capturing any other pathogen and bacteria	[189]
BC	Fe-Arg-MTX	IOMNPs	No side effects MRI contrast agent Effective and appropriate DDS for cancer cells Biocompatibility	[190]

Abbreviations: ALI—acute lung injury, BC—breast cancer, CPPs—cell-penetrating peptides, DDS—drug delivery system, Fe_3_O_4_ + TAT—TAT peptide conjugation to iron oxide NPs, Fe-Arg-MTX—arginine-coated Fe MNPs, GBM—glioblastoma multiforme, iNOS—inducible nitric oxide synthase, IOMNPs—iron oxide magnetic nanoparticles, IONPs—iron oxide nanoparticles, MRI—magnetic resonance imaging, NPs—nanoparticles, PNAs—peptide nucleic acids, pSiNRs—porous silicon nanorods, rGO—reduced graphene oxide, ROS—reactive oxygen species, SCKs—shell cross-linked knedel-like polymer nanoparticles, UTIs—urinary tract infections, VAN—vancomycin.

## 7. Clinical and Cost-Effectiveness Analysis of Application THPs with MNPs

To evaluate the clinical safety and cost-effectiveness of tumor-homing peptides functionalized with magnetic nanoparticles, we conducted research using two study registries: ClinicalTrials.gov [191] and the International Clinical Trials Registry Platform Search Portal [192]. The search strategy was prepared and conducted on March 24, 2024 according to the methodology outlined by the NIH—U.S. National Library of Medicine. We used the keywords “neoplasm”, “cancer”, “carcinoma”, “malignance”, “tumor”, and “tumour”, in collocations with “magnetic nanoparticles”, “MNPs”, “tumor homing peptides”, “THPs”, “cell penetrating peptides”, and “CPPs”. We adopted the following inclusion criteria: the research on the study topic, clinical trials with the “completed” status, and only specifying cancer cases related to the topic of the research, with results available. The cohort studies on the selected topic were excluded from the overall analysis. Based on the adopted inclusion and exclusion criteria, a total of four studies were eligible for a further clinical and cost-effectiveness analysis (Table 8).

Among four analyzed studies, only in one case [193] adverse events have been identified. The patients reported events in the following categories: cardiac disorder—palpitations (1); gastrointestinal disorders—vomiting (1); general disorder—catheter site rash (1); injury, poisoning, and procedural complications—thermal burn (1); musculoskeletal and connective tissue disorders—arthralgia (1); nervous system disorders—syncope, dizziness, sensory loss (3); psychiatric disorders—confusional state (1); skin and subcutaneous tissue disorders—pruritis (1). Additionally, no dose limiting toxicity was defined in this study.

## 8. Future Perspectives

Chemotherapy remains the most commonly used cancer treatment. Nevertheless, poor selectivity is an ongoing problem, which might be improved by using peptides with antitumor properties.

There is no doubt that tumor-homing peptides are attractive tools in cancer therapy, especially with carriers of therapeutic agents [197,198]. Furthermore, there has been an increasing focus on the identification of new peptide-based drug delivery systems with diverse payloads. The application of combined anticancer drugs can provide benefits such as improved therapeutic efficacy, enhanced chemical parameters like solubility and stability, reduced side effects, and lower costs of treatment [107,199,200].

Nowadays, there is a comprehensive research effort focused on the discovery of novel forms, such as peptides, peptoids, and peptidomimetics, that specifically target tumor cell surface receptors. These carriers, upon radiolabeling, can guide research into targeted diagnostics and therapeutics. Conclusively, modifying the surface of nanomaterials with different antitumor agents and peptides can increase cytotoxicity and selectivity against a growing number of malignancies [4,201].

Furthermore, the peptides discussed in this paper may be attractive as optimal drugs owing to their reduced production costs (peptide synthesis reagents that can support large amounts of reaction material for solid-phase peptide synthesis, greatly reducing costs), ability to be easily chemically modified, and high-tissue-penetration properties [202,203].

## 9. Conclusions

Recent decades indicated the great potential of functionalized magnetic nanomaterials for biomedical purposes and healthcare. A lot of studies demonstrated that magnetic nanoparticles have attracted attention in modern medicine and pharmacology owing to their potential usefulness as contrast agents for MRI, as colloidal mediators for cancer magnetic hyperthermia, or as active constituents of drug-delivery platforms. NPs have been shown to improve the pharmacokinetic profile of bio-molecular therapeutics by shielding them from rapid blood clearance or fast enzymatic destruction, resulting in safe drug delivery to the therapeutic area. In combination with CPPs, permeation into targeted cells is simplified, leading to greater intracellular carrier accumulation. Over the past few years, immense efforts have been devoted to develop and modify novel CPP sequences, demonstrating enhanced membrane permeability and improved targeted specificity. Additionally, the identification of shorter sequences is particularly valuable due to simplified synthesis pathways and reduced costs of production. Significantly, computational techniques are drawing much more attention to predict new CPP sequences.

Significant efforts have been made in the design of nanoplatforms decorated by homing molecules, which follow fundamental rules for therapeutic applications. These efforts have led to substantial developments in the field of nanomedicine, enabling the transport of therapeutic and/or diagnostic carriers across nearly impossible barriers such as the blood–brain barrier (BBB). Another challenge is to identify appropriate engineering methods to strictly control size and morphology to achieve better specificity and selectivity, and to gain an overview of the structural and environmental parameters relevant to their activity under physiological conditions. Furthermore, even though MNPs have found their way into clinics and the number of homing peptide-based clinical trials is growing, there are also some disadvantages of these conjugates. Among them are no long-term studies with the assessment of toxicity profiles of many nanomaterials, and the poor selectivity of many nano-based therapies can only be overcome by the functionalization of nanoparticles by homing peptides to enhance the targeting properties and improve the tumor penetration of these constructs.

The aging and mortality of the population as well as the need to introduce new medical technologies have economic implications, which result in increased spending on healthcare. These health-related expenditures are rising at an accelerated rate around the world, not only affecting governments but especially patients. There is no doubt that the introduction of new diagnostic and therapeutic solutions such as multifunctional nanoplatforms decorated by THPs, enabling a faster detection of cancer and its more effective treatment, will be reflected in the pharmacoeconomic aspect.

The examples from this paper about the use of THPs clearly indicated that the co-administration of anticancer drugs with the functionalized nanoforms could lead to remarkable improvement in anticancer treatment and its economic implications. The presented variety of strategies showed that the receptor specificity of drug-loaded nanoparticles can be enhanced using peptide-targeting ligands associated with cancer receptors. The synergism of nanoparticles with receptor-targeting peptides could result in the enrichment of their biofunctionality. All of the solutions discussed have an impact on the pharmacoeconomic aspect. Tumor growth inhibition through drug accumulation at the site of the lesion reduces direct needs (medical and non-medical) including hospitalization, adverse event management, non-medical services, and indirect costs of treatment processes such as reduced productivity at work, premature death, etc. Targeted and more efficient drug delivery leads to improvements in patient compliance as well as patients’ faster recovery, resulting in increased time and quality of life.

## Figures and Tables

**Table 1:** Bonding strategies of THP to nanoparticles.

Core Type	Av. Diameter	Interaction with THP	Application	Interaction with the Drug	Ref.
PEG polymer nanoparticles	20–50 nm	covalent; conjugation by NHS ester	siRNA delivery	non-covalent interactions	[49]
chemical complex	-	covalent; conjugation by maleimide	siRNA delivery	non-covalent interactions	[50]
PEG polymer nanoparticles	nd	covalent; conjugation by maleimide	adenovirus vector carrier	covalent (by maleimide or NHS ester)	[86,87,88,89,90,91]
PEG polymer nanoparticles	141–160 nm	covalent; Michael reaction	paclitaxel delivery	non-covalent interactions	[92]
PEG-PEI polymer nanoparticles	100 nm	covalent; conjugation by NHS ester	siRNA delivery	non-covalent interactions	[93]
PEG micelles	32 nm	covalent; reaction with acetal	siRNA delivery	covalent (by maleimide or NHS ester)	[94]
chitosan–PEG micelles	260 nm	covalent; esterification reaction	siRNA delivery	non-covalent interactions	[95]
PEG-PLA nanoparticles	120 nm	covalent; conjugation by maleimide	siRNA delivery	non-covalent interactions	[96]
mesoporous silica nanoparticles	51.2 nm	non-covalent interactions	doxorubicin and siRNA	non-covalent interactions	[97]
cationic liposome and PAA hybrid nanoparticles	93 nm	non-covalent interactions	siRNA delivery	non-covalent interactions	[98]
cyclodextrin	nd	non-covalent interactions	siRNA delivery	non-covalent interactions	[99]
pHPMA polyplexes	90–100 nm	covalent; amide bond formation	DNA delivery	non-covalent interactions	[100]
biomimetic magnetic nanoparticles@PLGA copolymer	220 nm	covalent; esterification reaction	drug delivery	nd	[101]

Abbreviations: PEG—Poly(ethylene glycol); pHPMA—Poly(N-(2-hydroxypropyl)methacrylamide); PEG-PLA—Polylactide-block-poly(ethylene glycol); PLGA—Poly (D, L-lactide-co-glycolide); PAA—Poly(acrylic acid); PEG-PEI—Poly(ethylene glycol)-block-polyethyleneimine.

**Table 2:** Peptide-conjugated nanocarriers and their benefits of use.

Core	Ligand/Vector	Agent	Effect	Ref.
Atelocollagen	Aptamer APT A10-3.2	miR15a, miR-16-1	reduction in damage to the bone tissue improvement in the therapeutic effect	[50]
PEG	Ac-YGGRGDTP(beta)A)(2)K-PEG-(beta)AC	Ads	modified adenovirus exhibited high gene expression even in a CAR-negative cell	[86]
PEG	RGD-Ads	dnIkappaB	gene therapy/tool in rheumatoid arthritis and in IBD therapy	[87]
Pluronic P85/PEI/TPGS	iRGD	PTX and survivin shRNA	powerful approach for the reversal and therapy of lung cancer resistance	[92]
PEG-b-PLL(2IT)	cRGD	SiRNAs	antiangiogenic therapy—suppression of angiogenesis and tumor growth rate	[94]
Chitosan–PEG	CP15 peptide	PLK1-siRNA	lack of systemic toxicity/potential approach for cancer therapy	[95]
Multi-layered nanocomplexes MNS/PAH-Cit/GTC	TAT peptide	DOX/VEGF-siRNA	strong anticancer effect	[97]
PLGA/lipid	iRGD	ICG/TPZ	delivery platform for PDT and hypoxia-activated chemotherapy	[63]

Abbreviations: PEG—poly(ethylene glycol); CP15—cell-targeting peptide; PLK1—serine/threonine-protein kinase; PAH-Cit—poly(allylamine hydrochloride)-citraconic anhydride; GTC—galactose-modified trimethyl chitosan–cysteine; VEGF—vascular endothelial growth factor; ICG—photosensitizer indocyanine green; TPZ—hypoxia-activated prodrug tirapazamine; PLGA—poly(D, L-lactide-co-glycolide); PEG-b-PLL(2IT)—poly(-b-PLL- poly(ethylene glycol)-block-poly(L-lysine) comprising lysine amines modified with 2-iminothiolane (2IT); cRGD—Cyclo-Arg-Gly-Asp peptide; PEI—polyethylenimine; TPGS—d-α-tocopheryl polyethylene glycol 1000 succinate; PTX—paclitaxel; DOX—doxorubicin;

**Table 3:** THPs for blood–brain barrier (BBB) delivery.

Peptide	Target	Agent	Ref.
Interleukin 13 peptide (IL-13p)	IL13Rα2	docetaxel	[119]
Tuftsin (TKPR)	Neuropilin-1 (NRP-1)	anthracyclines salicylanilides	[120,121,122]
tLyP-1	Neuropilin-1 (NRP-1)	5–carboxyfluorescein (FAM), ^18^F–fluoride	[123,124,125]
Azurin (Paz)	TKR	-	[126]
TGN	BBB AS1411 aptamer	docetaxel	[127,128]
Angiopep-2	LRP1	doxorubicin	[128]
Trans-activating transcriptional activator (TAT peptide)	Nucleus	siRNA expression plasmid, docetaxel, paclitaxel	[129,130]
Chlorotoxin (CTX)	Tumor cell surface receptor; MMP-2	platinum	[131,132]
BTP-7	dg-Bcan protein	camptothecin	[133]

**Table 8:** The clinical and cost-effectiveness benefits discussed in relation to THPs with MNPs.

**I. 68Ga-THP-PSMA PET/CT Imaging in High-Risk Primary Prostate Cancer or Biochemical Recurrence of Prostate Cancer (PRONOUNCED)** [193] **(49 Participants)** **Condition or Disease: Prostate Cancer**				
**Selected Study** **Details**	**Arms and Interventions**	**Brief Description**	**Clinical Analysis**	**Cost-Effectiveness Analysis**
**Official Study Title:** A Phase II, Open-label Study to Assess Safety and Clinical Utility of 68Ga-THP-PSMA PET/CT in Patients With High Risk Primary Prostate Cancer or Biochemical Recurrence After Radical Treatment (PRONOUNCED Study) **Study Phase:** 2 **Study** **Objectives:** Diagnostic **Study** **Design:** Interventional (Single Group Assignment), Open Label **Study Status:** Completed (June, 2019)	**Arm:** **1. Experimental:** Single i.v. administration of Gallium-68 THP-PSMA **Intervention/treatment:** **1. Drug:** Gallium-68 THP-PSMA (other name: THG-001)	**Brief Summary:** Open-labelled, single-center study in the UK The study group of 60 patients was divided into 3 smaller groups: - Group A: 20 patients newly diagnosed with primary high-risk prostate cancer and are scheduled for radical prostatectomy surgery - Group B: 20 patients with a diagnosis of BCR with previous radical prostatectomy, and are being considered for radical salvage therapy - Group C: 20 patients with a diagnosis of BCR with previous radical radiotherapy (no surgery) and are being considered for radical salvage therapy - From group A, B, and C, the actual enrollment was 49 patients	**Primary Outcome Measures:** **1. Change in Patient Management**—Measured as % of patients who had a change in management plan as a result of 68Ga-THP-PSMA PET/CT documented after scan, compared with their pre-scan management plan **Results:** No change (0% of 49 patients who underwent a technically successful post-baseline scan; the full analysis set and per protocol populations were the same) **Secondary Outcome Measures:** **1. Safety**—Treatment of Emergent AEs **Safety was assessed by** Physical examination Vital signs Cardiovascular profile Performance status Laboratory tests (hematology, biochemistry, urinary analysis, PSA) Recording of concurrent illness/therapy AEs **Results:** Mortality—0% SAEs—0% AEs—20%	**Increase in** LYG QALYs Five-year survival rate Progression-free time **Decrease in** DALYs No. of reported AEs No. of reported incidences of prostate cancer after implementation of 68Ga-THP-PSMA
**II. Pre-Operative Nodal Staging of Thyroid Cancer Using USPIO MRI: Preliminary Study** **(12 Participants)** [194] **Condition or Disease: Thyroid Cancer**				
**Selected Study** **Details**	**Arms and Interventions**	**Brief Description**	**Clinical Analysis**	**Cost-Effectiveness Analysis**
**Official Study** **Title:** Pre-Operative Nodal Staging of Thyroid Cancer Using Ultra-Small Superparamagnetic Iron Oxide Magnetic Resonance Imaging (USPIO MRI): Preliminary Study **Study Phase:** N/A **Study** **Objectives:** Diagnostic **Study** **Design:** Interventional (Single Group Assignment, Open Label) **Study Status:** Completed (April, 2016)	**Arm:** **1. Experimental:** Nanoparticle MRI Within 48–72 h after ferumoxytol infusion, a scan will be performed **Intervention/treatment:** **1. Drug:** Ferumoxytol i.v. administration at dose of 6 mg/kg of body weight, up to a maximum dose of 510 mg, delivered at a rate of up to 1 mL/s (other name: iron oxide-ferumoxytol) **2. Device:** Nanoparticle MRI within 48–72 h after i.v. ferumoxytol infusion, and a scan will be performed	**Brief Summary:** Open-labelled, single-center study in the USA (Massachusetts) Evaluation of the ability of USPIO MRI and ferumoxytol as experimental contrast agents to detect cancer in very small metastases in the thyroid lymph nodes	**Primary Outcome Measures:** Sensitivity of LSN MRI Specificity of LSN MRI **Results:** Mortality—0% SAEs—0% AEs—0%	**Increase in** No. of early detected cancer metastases
**III. Clinical and Technical Feasibility of an Ultrasuperparamagnetic Nanoparticle Iron Oxide (USPIO)-Enhanced** **Magnetic Resonance Lymph Node Imaging** [195] **(10 Participants)** **Condition or Disease: Cancer of Lymph Nodes**				
**Selected Study** **Details**	**Arms and Interventions**	**Brief Description**	**Clinical Analysis**	**Cost-Effectiveness Analysis**
**Official Study Title:** Clinical and Technical Feasibility of a Ultrasuperparamagnetic Nanoparticle Iron Oxide (USPIO)-Enhanced Magnetic Resonance Lymph Node Imaging **Study Phase:** N/A **Study** **Objectives:** Diagnostic **Study Design:** Interventional (Single Group Assignment), Open Label **Study Status:** Completed (July 2019)	**Arm:** **1. Experimental:** Feraheme MRI within 48–72 h after i.v. administration of Feraheme^®^ **Intervention/treatment:** **1. Drug:** Feraheme i.v. administration at dose of 6 mg of iron/kg (maximum: 510 mg/dose) at a rate of 1 mL/s (30 mg/s) or slower after initial MRI (other name: ferumoxytole) **2. Procedure**: MRI MRI scan performed: Before i.v. administration of Feraheme; After i.v. administration of Feraheme, MRI scan performed 2 days later, and then again the following day.	**Brief Summary:** Open-labelled, single-center study in the USA (Texas) Evaluation of the ability of USPIO MRI and Feraheme® (ferumoxytole) as experimental contrast agents to detect cancer in very small metastases in the cancer of lymph nodes as well as in liver imaging	**Primary Outcome Measures:** 1. No. of patients with SI change in a lymph node—comparison between the pre- and post-contrast **Results:** Mortality—0% SAEs—0% AEs—0%	**Increase in** No. of early detected cancer metastases **Change in** Optimum scan time due to SI changes in a lymph node
**IV. Pre-Operative Staging of Pancreatic Cancer Using Superparamagnetic Iron Oxide Magnetic Resonance Imaging (SPIO MRI)** [196] **(35 Participants)** **Condition or Disease: Pancreatic Cancer**				
**Selected Study** **Details**	**Arms and Interventions**	**Brief Description**	**Clinical Analysis**	**Cost-Effectiveness Analysis**
**Official Study Title:** Improved Pre-Operative Staging of Pancreatic Cancer Using Superparamagnetic Iron Oxide Magnetic Resonance Imaging (SPIO MRI) **Study Phase:** 4 **Study** **Objectives:** Diagnostic **Study Design:** Interventional (Single Group Assignment), Open Label **Study Status:** Completed (February 2013)	**Arm:** **1. Experimental:** SPIO MRI **Intervention/treatment:** **1. Drug:** SPIO MRI Two MRIs will be performed over a 2-day period. The second scan will be performed 48 h after i.v. administration of ferumoxytol (other names: SPIO MRI, USPIO, feruoxytol)	**Brief Summary:** Open-labelled, single-center study in the USA (Massachusetts) Evaluation of the ability of USPIO MRI and ferumoxytol as experimental contrast agents to identify small and otherwise undetectable lymph node metastases in patients with pancreatic cancer who are scheduled for surgical resection	**Primary Outcome Measures:** Sensitivity of LSN MRI Specificity of LSN MRI **Results:** Mortality—0% SAEs—0% AEs—0%	**Increase in** No. of early detected cancer metastases

Abbreviations: AEs—Adverse events, BCR—Breakpoint cluster region; type of gene, DALYs—Disability-adjusted life years, Gallium-68 THP-PSMA = THG-001—Name of drug, LSN MRI—Magnetic resonance imaging with lymphotrophic superparamagnetic nanoparticles, LYG—Life years gained, MRI—Magnetic resonance imaging, N/A—Not applicable, No.—Number, PET/CT—Positron emission tomography/computed tomography, SAEs—Serious adverse events, SI—Signal intensity, SPIO MRI—Superparamagnetic iron oxide magnetic resonance imaging, UK—The United Kingdom, QALYs—Quality-adjusted life years.
